# How to Select Firefly Luciferin Analogues for In Vivo Imaging

**DOI:** 10.3390/ijms22041848

**Published:** 2021-02-12

**Authors:** Ryohei Saito-Moriya, Jun Nakayama, Genta Kamiya, Nobuo Kitada, Rika Obata, Shojiro A. Maki, Hiroshi Aoyama

**Affiliations:** 1 School of Pharmacy, Tokyo University of Pharmacy and Life Sciences, Tokyo 192-0392, Japan; aoyamahr@toyaku.ac.jp; 2 Department of Engineering Science, Graduate School of Informatics and Engineering, The University of Electro-Communications, Tokyo 182-8585, Japan; kamiya0801@uec.ac.jp (G.K.); kitada@uec.ac.jp (N.K.); robatad@uec.ac.jp (R.O.); s-maki@uec.ac.jp (S.A.M.); 3 Center for Neuroscience and Biomedical Engineering, The University of Electro-Communications, Tokyo 182-8585, Japan; 4 Division of Cellular Signaling, National Cancer Center Research Institute, Tokyo 104-0045, Japan; jnakayama.re@gmail.com

**Keywords:** bioluminescence imaging, near-infrared light, luciferin analogue, luciferase, multicolor, high sensitivity

## Abstract

Bioluminescence reactions are widely applied in optical in vivo imaging in the life science and medical fields. Such reactions produce light upon the oxidation of a luciferin (substrate) catalyzed by a luciferase (enzyme), and this bioluminescence enables the quantification of tumor cells and gene expression in animal models. Many researchers have developed single-color or multicolor bioluminescence systems based on artificial luciferin analogues and/or luciferase mutants, for application in vivo bioluminescence imaging (BLI). In the current review, we focus on the characteristics of firefly BLI technology and discuss the development of luciferin analogues for high-resolution in vivo BLI. In addition, we discuss the novel luciferin analogues TokeOni and seMpai, which show potential as high-sensitivity in vivo BLI reagents.

## 1. Introduction

There are many bioluminescent organisms [1,2,3], such as the firefly and the luminous shrimp. These organisms produce light by bioluminescence reactions involving the oxidation of a luciferin (substrate) catalyzed by a luciferase (enzyme) (Table 1). For instance, firefly bioluminescence originates from the oxidation of *d*-luciferin (DLH2) catalyzed by firefly luciferase (Fluc) in the presence of adenosine triphosphate (ATP), Mg^2+^ and O_2_. This reaction proceeds by two-steps: DLH2 is first adenylated with ATP, and then it is oxidized by O_2_, to generate excited oxyluciferin, which produces yellow-green light (λ_max_ = 560 nm) [4,5,6,7]. In contrast, bioluminescence in the luminous shrimp, one of many bioluminescent marine species, is generated by the oxidation of coelenterazine (CTZ) catalyzed by *oplophorus* luciferase (Oluc) in the presence of O_2_ to generate coelenteramide, which produces blue light (λ_max_ = 454 nm) [8,9,10]. 

Recently, Oba et al. published a report on the evolutionary history of firefly bioluminescence [11]. They recreated putative ancestral firefly luciferases by predicting their amino acid sequences with a maximum-likelihood method of ancestral state reconstruction [12] and experimentally characterized their enzymatic properties, including their luminescence colors. The firefly family evolved from Lampyridae–Phengodidae–Rhagophthalmidae lineage, which produces a weak and red light, with the first firefly that appeared producing deep-green light. Thereafter, fireflies with different luminescent colors appeared gradually, as the species underwent divergent evolution. These results suggest to be extremely useful information in the case of improving luciferase mutants for changing bioluminescence colors in the future.

Firefly luciferin- and luciferase-based bioluminescence imaging (BLI) is widely utilized in the life science and medical science fields [13,14], for in vitro and in vivo study of the central nervous system (CNS) [15], fungal pathogens [16,17], immunity [18], microglia [19], neuroinflammation [20], parasites [21], stem cells [22], transporters [23], and viruses [24,25,26], among others. For example, in vitro luciferase assays are widely used for the quantification of gene promoter activities [27]. Luciferin–luciferase reactions are also used for in vivo real-time monitoring of gene expression, cell numbers, and other biological events. Specifically, firefly BLI has been utilized for in vivo study in numerous mouse models [28,29,30,31,32,33,34,35,36]. For instance, it has been used to quantify tumor cells [37] and monitor gene expression in transgenic mouse models [38]. Now many researchers use the improved luciferase genes for the experiment in life science (e.g., *luc+*, *luc2*). The *luc2* gene, which is developed by adjustment of codon and reducing of the transcription factor binding site from wild-type luciferase sequence, enables the high expression of luciferase in mammalian cells [39].

On the other hand, many researchers have synthesized CTZ analogues for application to optical imaging [40,41,42,43,44,45,46,47,48]. For instance, Hall et al. developed an artificial bioluminescence pair, the substrate furimazine (FMZ) and the mutant luciferase NanoLuc^®^ [49,50,51]. Since the FMZ/NanoLuc^®^ reaction is independent of the concentration of ATP [52], it is a useful tool for studying exosomes, blood, and urine, where ATP abundance is limited [53]. This is particularly important because the native firefly bioluminescence reaction is not suitable for monitoring exosomes [54], as it is ATP-dependent. However, generally, CTZ and derivatives are proceeded by auto-oxidation, in the absence of luciferase, resulting high background signals are detected in cell or animal experiments [55]. In contrast, DLH2 has some advantages, such as low background signals, excellent bioavailability, and easy handling [56].

Firefly BLI methods are based on the detection of optical signals and are, thus, more suitable for imaging shallow tissues, such as subcutaneous tissue, than for imaging deeper tissues, such as those in the brain or lungs [57,58]. This is because deep-tissue imaging is difficult by the poor light permeability of biological tissue. Yellow-green light, produced by natural firefly bioluminescence (DLH2/Fluc), is absorbed and scattered by bio-tissue [59,60]. Therefore, BLI of DLH2/Fluc suffered from weak luminescence intensity and short emission wavelengths (560 nm). Another reason is the poor biodistribution of DLH2 [61]. For example, a study using ^14^C labeled DLH2 revealed that, after 15 min of intraperitoneal administration, DLH2 is distributed mainly at the skin and does not reach the brain or lungs [62].

Accordingly, numerous luciferin analogues [63,64] and mutant luciferases [65,66,67,68,69,70] have been developed for high-resolution detection. A particularly important development in in vivo BLI technology is the development of luciferin analogues and mutant luciferases that provide near-infrared (NIR) wavelength luminescence. This is because NIR imaging makes it possible to efficiently image deep tissue even at lower luminescence intensity.

Since different luciferin analogues and luciferase mutants have their own strengths and weaknesses, it is important to choose the right combination for a particular in vitro and in vivo experiment. Most previous research has used single-color in vivo BLI. However, multicolor bioluminescence reactions have been developed for application to in vivo imaging.

In this review, we present a brief introduction to the characteristics of firefly BLI technology and discuss advances in the development of luciferin analogues and luciferase mutants for high-resolution in vivo BLI.

## 2. The Development of Luciferin and Luciferase for NIR BLI

### 2.1. Luciferin Analogues that Produce NIR Light

Luciferin analogues have been synthesized as a means to overcome the two weaknesses of DLH2 mentioned above, i.e., low light permeability and biodistribution, thus realizing higher sensitivity and utility for in vivo imaging (Table 2). A viable strategy here is to change the luminescence color of DLH2 from yellow-green to NIR light. Since NIR light permeates biological tissues well, NIR luminescence is suitable for detecting signals from deep tissue, such as the brain and lungs [59]. Another strategy is the synthesis of luciferin analogues with increased hydrophobicity for improved cell membrane permeability [71]. These two approaches have resulted in luciferin analogues with improved characteristics for in vivo imaging, as discussed below.

Miller et al. developed CycLuc1, in which a pyrrolidine moiety is fused to the benzothiazole moiety of DLH2. The wavelength of CycLuc1 is 599 nm (*K*_m_ = 0.10 and 1.06 µM in Table 2), which is longer than that of DLH2 due to electron donation from the fused *N*-hetero ring [75]. The intensity of CycLuc1 BLI in the brain striatum is eight-times more than that of DLH2 [57].

Similarly, Li et al. developed CybLuc, in which the hydroxy group of DLH2 is replaced with an *N*-cycloalkyl group, shifting the wavelength from that of DLH2 to 603 nm (*K*_m_ = 0.16 µM in Table 2). The intensity of CybLuc BLI in the brain hippocampus is approximately 18-times higher than that of DLH2 [73]. Furthermore, since the hydrophobicity of these luciferin analogues is higher than that of DLH2, their blood–brain barrier (BBB) permeability is improved, resulting in higher BLI intensity in deep-brain tissues.

In contrast, Iwano et al. developed AkaLumine, in which only the thiazoline moiety of DLH2 is retained. The benzothiazole moiety is substituted with a simple benzene ring, and the π-conjugation of the molecule is extended with olefins [84]. The wavelength of AkaLumine is 675 nm (*K*_m_ = 0.195–2.78 µM in Table 2), which shows in the NIR region, and its hydrophobicity is improved, resulting in higher cell-membrane permeability. However, AkaLumine is not soluble in phosphate buffered saline (PBS) or ultra-pure water to the amount needed for administration to animals. This is an example of the trade-off relationship between cell-membrane permeability and solubility for administration. Accordingly, in order to allow better water solubility than that of AkaLumine, the related luciferin analogues TokeOni and seMpai were developed [58,82]. These analogues are discussed in more detail in Section 3.

Moreover, Ikeda et al. synthesized NIRLuc2 based on AkaLumine, with a fused *N*-hetero ring (like CycLuc1). NIRLuc2 exhibits wavelength at 683 nm (*K*_m_ = 1.05 µM in Table 2) [83]. However, unlike AkaLumine, NIRLuc2 is soluble in PBS (-) containing 5% DMSO. The authors then performed BLI, using NIRLuc2 on a subcutaneous tumor mouse model after intraperitoneal administration, revealing that the luminescence intensity of NIRLuc2 is approximately seven-fold that of DLH2 and similar to that of AkaLumine [83]. 

Extending the π-conjugation of DLH2 is a valid strategy for obtaining analogues with wavelengths in the NIR region. Using this strategy, Anderson et al. developed infraluciferin (iLH2), in which the π-conjugation is extended with an olefin moiety [90]. The wavelength of iLH2 upon reaction with a S284T mutant Fluc is 706 nm (*K*_m_ = 6.0 µM with x5 Flu mutant in Table 2). The same authors have also developed an in vivo dual-imaging technique that combines iLH2 and DLH2 with two different enzymes, presenting the possibility of observing new biological events by tracking two processes simultaneously [91]. In addition, Hall et al. synthesized NH_2_-NpLH2, in which the *π*-conjugation of the benzothiazole moiety in DLH2 is extended by fusion with an aromatic ring at the 6’- and 7’- positions [74]. NH_2_-NpLH2 reacts with CBR2opt, which is a mutant luciferase based on click beetle luciferase, to produced light with a wavelength of 743 nm (*K*_m_ = 0.5 µM in Table 2). BLI of mice, using NH_2_-NpLH2/CBR2opt, is not significantly improved compared to those using DLH2/CBR2opt or TokeOni/Luc2. However, since there is no other bioluminescence reaction with a wavelength as long as 743 nm, the application of NH_2_-NpLH2 could be expanded by employing it in combination with an NIR-fluorescent protein for BRET imaging.

### 2.2. The Development and Evaluation of Combinations of Luciferin Analogues and Luciferase Mutants

Zambito et al. investigated the bioluminescence characteristics of each possible combination of luciferin (DLH2, CycLuc1, NH_2_-NpLH2, or TokeOni) and luciferase (Luc2, CBR2, CBG99, or AkaLuc) [93]. The bioluminescence intensities and time-course of each combination were found to be different, indicating that there is an optimal luciferase for each substrate. The highest luminescence intensity for each luciferin, the luciferase used, and the time point at which it was presented were reported to be DLH2/Luc2 at 10 min, CycLuc1/AkaLuc at 10 min, NH_2_-NpLH2/CBR2 at 20 min, and TokeOni/AkaLuc at 10 or 15 min. Additionally, they measured the bioluminescence wavelength of each combination in vivo, using bandpass filters. Interestingly, the wavelength was very different for each combination. That for DLH2/Luc2 was 610 nm, DLH2/CBG99 was 540 nm, NH_2_-NpLH2/CBR2 was 730 nm, and NH_2_-NpLH2/CBG99 was 620 nm. Each combination for DLH2 and NH_2_-NpLH2 shifts by approximately 100 nm, depending on the luciferase. Conversely, the wavelength for CycLuc1/AkaLuc was 600 nm, and that for TokeOni/AkaLuc was 660 nm. Although the wavelengths for these two substrates change with different luciferases, the degree of the shift is approximately 20–40 nm. Thus, the combination of luciferin and luciferase is very important, and if the right combination is not used, it will be difficult to detect the observed tissues with high sensitivity.

**Figure 1:**
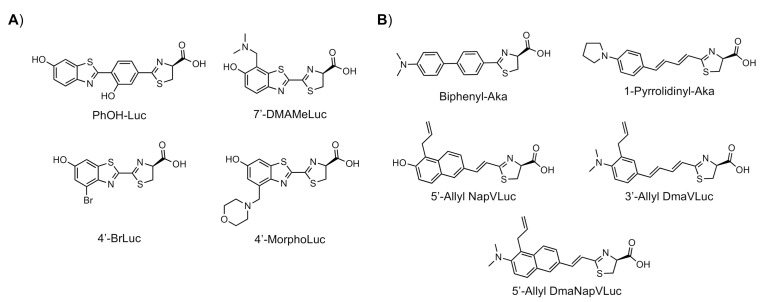
The structures of several artificial bioluminescence substrates (**A**) and weak-intensity luciferin analogues (**B**).

These results demonstrate that bioluminescence intensity and wavelength can be changed significantly by developing luciferase mutants specialized for luciferin analogues. Furthermore, orthogonal combinations with characteristic properties can be expanded the variation of applications. For instance, Prescher et al. developed orthogonal combinations of luciferin analogues and luciferase mutants [94,95,96]. They reported that the bioluminescence of PhOH-Luc (Figure 1A) with Fluc is weak. However, it is improved by using a mutant luciferase named G4 in which the amino residues near the active site were restructured, using Rosetta software [97,98,99]. The resulting PhOH-Luc/G4 pair was found to exhibit better bioluminescence activity than the PhOH-Luc/Fluc pair. Using the same method, they developed three different specialized mutants, such as mut95, mut53, and mut81, that adapts to each of the substrates 7′-DMAMeLuc, 4′-BrLuc, and 4′-MorphoLuc (Figure 1A), respectively [94]. These unique luciferin–luciferase pairs enabled orthogonal triplet and quartet imaging to be readily achieved. In the future, this kind of multiple artificial bioluminescence may enable multicomponent imaging.

Maki et al. reported a series of luciferin analogues (Figure 1B) that react with Fluc and to produce NIR light [100,101,102,103], and several of them were evaluated for BLI, using a mouse model. However, these analogues exhibit much lower luminescence intensities than TokeOni. Nevertheless, if a high-orthogonality pair can be realized by developing a specialized luciferase for analogue, using a software-based mutation methodology similar to that employed by Prescher et al., it may be suitable for successful application in vivo.

Indeed, dual-color BLI technology has already been explored in some animal studies. For instance, Aswendt et al. performed dual-color in vivo BLI of mouse brain tissues, allowing them to monitor both stem cell survival and differentiation in one imaging session simultaneously [104]. Furthermore, Doi et al. monitored the expression profiles of two different genes in *Caenorhabditis elegans*, using a dual-luciferase system based on Eluc (Emerald luciferase) and SLR (stable luciferase red) [105]. Dual/multi-color BLI system enables us to monitor several events, such as several genes’ expression and cellular events. Most of the previous research detected a single biological event, using single-color BLI. Thus, multicolor BLI may help to discover complex biological event in the future.

## 3. Chemical and Physical Characteristics of the NIR Luciferin Analogues TokeOni and seMpai

### 3.1. Development of TokeOni (AkaLumine-HCl)

The solubilities of AkaLumine in PBS and ultra-pure water are very poor, and thus usability was limited for in vivo imaging [58,83]. Therefore, TokeOni [58] and seMpai [82], the hydrochloric acid salt of AkaLumine and N-atom containing AkaLumine derivative, respectively, were prepared with the aim of improving water solubility. The wavelength from TokeOni upon reaction with Fluc occurs at 677 nm, similar to that of AkaLumine (675 nm) [58]. However, the water solubility of AkaLumine is 2 mM in ultra-pure water, while that of TokeOni is 40 mM, which is 20-fold higher [58]. This makes it possible to administer the reagent with a smaller solution volume and a higher concentration than Akalumine. Furthermore, the intensity of BLI in the lung tissue of mice achieved using TokeOni is significantly higher than that achieved with DLH2 and CycLuc1. However, the luminescence intensity of TokeOni/Fluc is not sufficient for imaging in large animal models, such as marmosets.

Accordingly, Iwano et al. developed a mutant luciferase named AkaLuc, which was tailored for TokeOni, and termed their new TokeOni/AkaLuc combination method as AkaBLI [81]. The intensity of AkaBLI (TokeOni/AkaLuc) is approximately 10-fold higher in cells, 52-fold higher in mouse lung, and 1400-fold higher in mouse brain tissue than that obtained with DLH2/Fluc. In addition, AkaBLI was able to detect single mouse lung cell and to quantify 1–10 cells. Moreover, AkaBLI with video-rate was able to monitor signals from the brain striatum in freely moving mice and common marmoset. Although AkaBLI was sufficiently utilized for deep-tissue imaging, this system was not enabled to quantitatively detect signals from exosomes [54]. This result suggests the need for new luciferin analogues that produce sufficient light, even under low-ATP-concentration conditions.

### 3.2. Development of seMpai

Two characteristics of TokeOni were improved by seMpai; since the pH value of TokeOni is acidic [89], acidosis is a potential problem, and hepatic background signals are detected when using TokeOni [89, 106] but not when using DLH2. Furthermore, seMpai is a luciferin analogue designed with containing N-atom in the AkaLumine moiety, slightly changing its bioluminescence reaction with Fluc and AkaLuc (*K*_m_ = 6.2 and 44.9 µM, respectively in Table 2). Moreover, seMpai allows high-sensitivity detection in in vivo BLI, and it can be used to detect pulmonary micro-metastases. In addition, seMpai is highly soluble (69 mM) in PBS (pH 7.4) [82] and does not produce hepatic background signals in breast cancer metastasis models [89]. However, seMpai BLI cannot detect biological events at single-cell-level resolutions, like AkaBLI. Thus, the future development of a novel mutant luciferase specifically for seMpai may realize single-cell-level resolution imaging in vivo. Interestingly, the in vivo dynamics of seMpai are similar to those of DLH2 [86], as described below.

### 3.3. Differences between Luciferin Analogue Dynamics In Vivo

Recent studies have provided important information for the selection of in vivo BLI systems. For instance, Fukuchi et al. performed a comparative analysis of DLH2, TokeOni and seMpai by monitoring the expression of brain-derived neurotrophic factor (BDNF) in Bdnf-luc transgenic mice. They reported the interesting finding that TokeOni BLI shows a different luminescence than those for DLH2 and seMpai, which show the same pattern in brain BLI [86]. Furthermore, TokeOni is more useful than seMpai for brain BLI [81,86], as it exhibits a higher BBB permeability than seMpai, due to its higher hydrophobicity.

**Figure 2:**
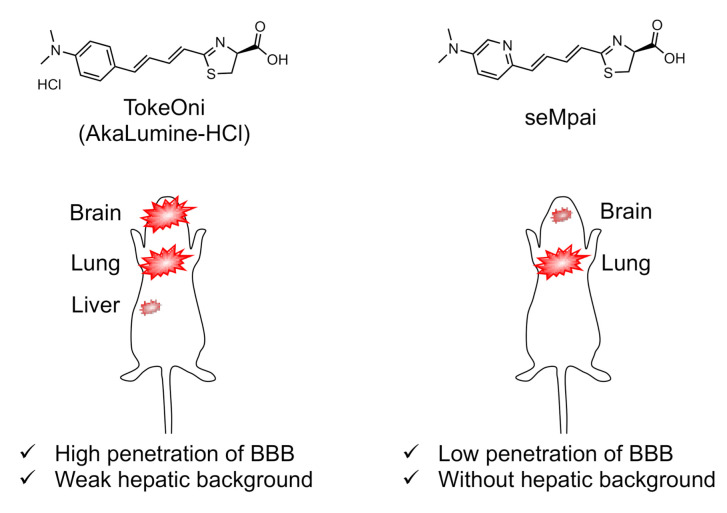
The different bioluminescence imaging (BLI) activities of TokeOni and seMpai and their structures. TokeOni enables high-resolution imaging of brain and lung tissues at the single-cell level but presents hepatic background signals, which is caused by its hydrophobicity; however, seMpai enables high-resolution BLI without hepatic background signals, but its hydrophilicity interferes its penetration of the blood–brain barrier (BBB).

The fact that bioluminescence patterns change depending on the tissue permeability of the luciferin analogues used is a serious problem because it means that the chemical and physical properties of luciferin analogues have significant effects on biological discovery. Nevertheless, since seMpai BLI reduces hepatic background signals, it is very useful for the study of systemic mouse models, such as those used to study metastasis [89] (Figure 2). Thus, it is important that researchers fully understand the characteristics of their chosen in vivo BLI system when applying it to their studies. There are very few reported studies that focus on comparative analysis of the in vivo dynamics and kinetics of luciferin analogues based on their physicochemical properties. Thus, it is necessary that the appropriate luminescence tools be determined according to the organ(s) to be observed.

## 4. Conclusions

In this review, we have summarized the development and application of luciferin analogues for in vivo BLI and NIR imaging technology. We suggest that the development of luciferin analogues should not just focus on bioluminescence activity, but also consider improved pharmacokinetics for animal safety. The informed combination of luciferin analogue and mutant luciferase can be used to tailor luminescence wavelength and intensity, as well as dynamics in vivo. Thus, researchers using such techniques for life science and medical research must be aware of the influence of BLI system selection.

## Figures and Tables

**Table 1:** Details of typical natural substrates and co-factors for bioluminescence reactions.

Scheme	Luciferase	Species	Co-Factors	References
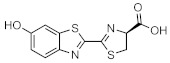 *d*-Luciferin (DLH2)	Firefly luciferase (Fluc)	*Photinus pyralis*	ATP, O_2_, Mg^2+^	[1,2,3,4,5,6,7]
	Click beetleluciferase (CBluc)	*Pyrophorus* *plagiophthalamus*		
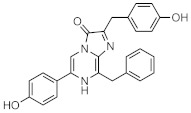 Coelenterazine (CTZ)	*Gaussia* luciferase (Gluc)	*Gaussia princeps*	O_2_	[1,2,3,8,9,10]
	*Renilla* luciferase (Rluc)	*Renilla reniformas*		
	*Oplophorus* luciferase (Oluc)	*Oplophorus* *gracilirostris*		

**Table 2:** Application of firefly bioluminescence reactions to imaging.

Substrate	Luciferase	Wavelength	*K*_m_^1^	Relative Intensity ^2^	Target Organ	References
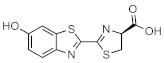 *d*-Luciferin(DLH2)	Firefly luciferase (luc, luc+, luc2)	560 nm	107 µM [58], 6.76 µM [72], 22.10 µM [73], 1 µM [74]	1	bone, brain, breast, lung, small intestine, subcutaneous	[15,16,18,19,20,21,24,28,29,30,31,32,33,34,35,36,37]
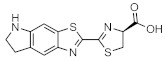 CycLuc1	Firefly luciferase (luc, luc+, luc2)	599 nm	1.06 µM [58] 0.10 µM [72]	0.7 (in vitro) 8 (brain)	brain	[57,75,76,77,78,79,80]
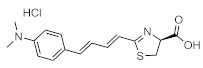 TokeOni (AkaLumine-HCl)	Firefly luciferase (luc, luc+, luc2)	675 nm	2.06 µM [58], 2.78 µM [81], 1.3 µM [82], 0.195 µM [83]	0.05 (in vitro) 40 (subcutaneous) 8.1 (lung)	brain, lung, subcutaneous	[22,54,58,81,84,85,86,87,88]
	AkaLuc (mutant)	650 nm	8.96 µM [81], 4.55 µM [89]	10 (in cell) 1408 (brain) 52 (lung)		
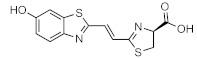 Infraluciferin (iLH_2_)	Fluc_red S284T Fluc (mutant)	706 nm	6.0 µM [90] ^4^	7.28 (intracranial) 4.1 (systemic) 42 (subcutaneous)	brain, lymphoma, subcutaneous	[90,91]
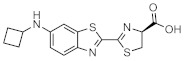 CybLuc	Firefly luciferase (luc, luc+, luc2)	603 nm	0.16 µM [73]	18 (brain) 20 (subcutaneous)	brain, subcutaneous	[73,92]
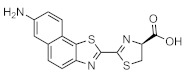 NH_2_-NpLH2	Firefly luciferase (luc, luc+, luc2)	No emission	–	–	brain, subcutaneous	[74]
	CBR2opt (mutant)	743 nm	0.5 µM [74]	0.3 (brain) 0.5 (subcutaneous)		
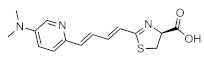 seMpai	Firefly luciferase (luc, luc+, luc2)	675 nm	6.2 µM [82]	0.1 (in vitro) 0.7 (brain) 6 (lung) 1 (subcutaneous)	brain, breast, lung, subcutaneous	[82,86,89]
	AkaLuc (mutant)	640 nm	44.9 µM [89]	0.2 (in vitro) ^3^		
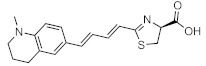 NIRLuc2	Firefly luciferase (luc, luc+, luc2)	683 nm	1.05 µM [83]	7 (subcutaneous)	subcutaneous	[83]

^1^ The detailed conditions are various and confirm if need. ^2^ Relative light intensity compared to DLH2. ^3^ Relative light intensity compared to TokeOni/AkaLuc. ^4^ The *K*_m_ value with x5 Fluc, which is a thermostable Fluc mutant.

## Abbreviations

ATP	Adenosine triphosphate
BBB	Blood–brain barrier
BDNF	Brain-derived neurotrophic factor
BLI	Bioluminescence imaging
CBR	Click beetle luciferase
CTZ	Coelenterazine
DLH2	*d*-Luciferin
Eluc	Emerald luciferase
Fluc	Firefly luciferase
FMZ	Furimazine
FMZ	Furimazine
Gluc	*Gaussia* luciferase
iLH2	Infraluciferin
NIR	Near-infrared
Oluc	*Oplophorus* luciferase
PBS	Phosphate buffered saline
Rluc	*Renilla* luciferase
SLR	Stable luciferase red
